# A tunable synthesis of indigoids: targeting indirubin through temperature†

**DOI:** 10.1039/d2ra00400c

**Published:** 2022-02-15

**Authors:** James A. Shriver, Kaylie S. Kaller, Ally L. Kinsey, Katelyn R. Wang, Summer R. Sterrenberg, Madison K. Van Vors, Joshua T. Cheek, John S. Horner

**Affiliations:** Central College 812 University St. Campus Box 020 Pella IA 50219 USA shriverj@central.edu

## Abstract

The spontaneous conversion of 3-indoxyl to indigo is a well-established process used to produce indigo dyes. It was recently shown that some indoles, when reacted with molybdenum hexacarbonyl and cumyl peroxide, proceed through an indoxyl intermediate to produce significant amounts of indirubin through a competing mechanism. Modulation of this system to lower temperatures allows for careful tuning, leading to selective production of indirubins in a general process. A systematic assay of indoles show that electron deficient indoles work well when substituted at the 5 and 7 positions. In contrast, 6-substituted electron rich indoles give the best results whereas halogeno indoles work well in all cases. This process shows broad functional group tolerance for generally reactive carbonyl-containing compounds such as aldehydes and carboxylic acids.

## Abstract 2

Selectivity of indigoid synthesis shifts from indigo to indirubin at reduced temperatures across a range of substrates.

## Graphical abstract

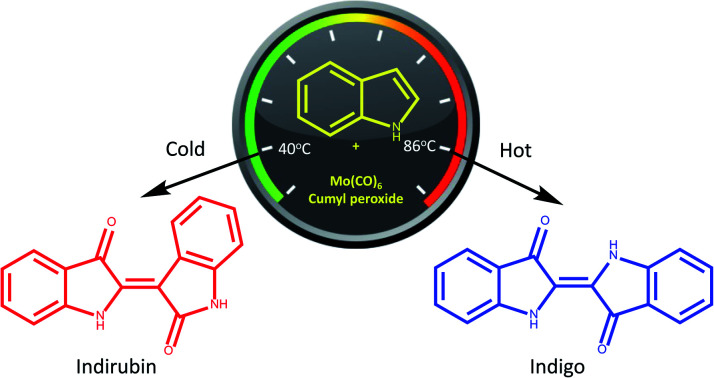

## Introduction

The investigation of indirubin (1) for its potential as a kinase inhibitor has been ongoing, leading to a steady influx of studies over the past 25 years. It was identified as the active component of Danggui Longhui Wan, a traditional Chinese medicinal recipe used to treat Chronic Myelocytic Leukemia (CML).^1^ Within the last two years, research has been conducted related to, *inter alia*, insulin resistance,^2^ paraptosis in MDA-MB-231 breast cancer cells,^3^ anti-tumor effects in Thyroid Cancer,^4^ and inhibition of osteosarcoma cells.^5^ A comprehensive review and meta-analysis related to anticancer applications was recently published and demonstrates the potential for the development of selective indirubin moieties.^6^ Beyond medical applications, indirubin was recently shown to exhibit photoswitching behavior with red light, which is enhanced when stabilized by supramolecular interactions with a thiourea additive thus commencing a new line of inquiry for this molecule.^7^

**Fig. 1:**
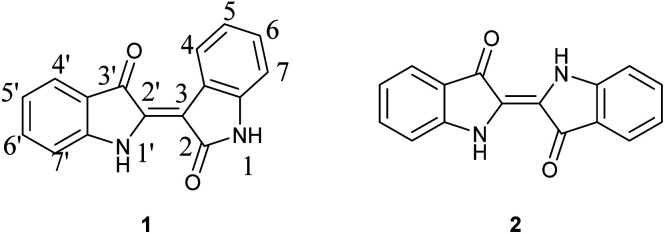
Chemical structures for indirubin (1) and indigo (2) with accepted numeric annotations indicated.

While the applications of indirubins is ongoing in the research community, the development of new synthetic approaches has lagged despite the relatively simple structure. As indicated in Fig. 1, indirubin is a structural isomer of indigo (2), where the connection occurs between the 3 and 2′ positions of indole rings, as opposed to the 2 and 2′ positions. The two compounds are easily distinguished by UV/Vis-different indigos give a maximum absorbance at approximately 590–640 nm, whereas indirubin's maximum absorbance occurs between 500–560 nm, depending on substituents and their location. Likewise, indirubins can be distinguished by NMR techniques due to their difference in symmetry and a unique downfield absorbance for the hydrogen located at the 4-position on indirubin.^8^

**Scheme 1:**
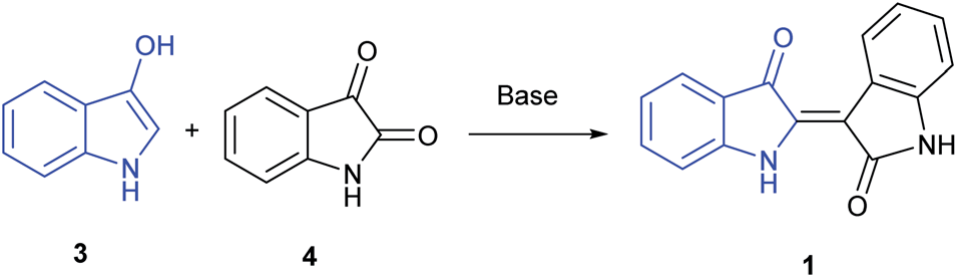
Baeyer procedure for the reaction between 3 and 4.

A majority of indirubin syntheses utilize the venerable Baeyer method,^9^ developed in the 1880's, that involves the base-catalysed condensation of 3-indoxyl (3), generated *in situ*, with isatin (4), as shown in Scheme 1, with the indoxyl-derived component highlighted in blue. A limitation of this method is the convenient access to indoxyl precursors, which are derived from precursors that are hard to access in large quantities beyond the parent substrate. These include indican, the 3-indoxyl-B-glucanopyroside used as a precursor to indigo, or 3-indoxyl acetate, which can be synthesized in small quantities from indoles using a hypervalent (diacetoxyiodo)benzene reagent.^10^ Given the ubiquitous nature of indigo in society, it is unsurprising that more contemporary synthetic methods are dominated by biologically inspired enzymatic approaches. Examples include explorations by Maugard implicating the presence of an isatin precursor^11^ and the utilization of cytochrome P450 enzymes.^12^

In contrast to the biologically derived methods, there are few viable examples that utilize traditional benchtop chemistry that operate at significant scale with indirubin often produced as a by-product. One example is for the use of Oxone® with 3-formyl indoles which produces indirubin as a side product.^13^ The most notable and direct comparison to the work in this publication is related to the reductive dimerization of isatin using potassium borohydride.^14^ While only applicable to symmetrically substituted systems not prone to reduction, the scale of the reactions (10 mmol) and yields (63–91%) show potential for broader-scale use.

A unique synthesis for 7,7′-diazaindirubin was recently published by this group employing an indoxyl intermediate independently generated through two methods.^15^ The first approach utilized a molybdenum-catalysed oxidation of the corresponding 7-azaindole through a method initially developed by Yamamoto and co-workers to synthesize indigo from indole.^16^ The second approach used 7-azaindoxyl acetate as a precursor to produce 7,7′-diazaindirubin using acid-catalysed hydrolysis with either trichloroacetic acid or ytterbium triflate with significant heat in the presence of oxygen. Unlike other studies proceeding through an indoxyl intermediate, where indigo is the predominant and often sole product, indirubin was produced exclusively for this substrate. Utilizing the Yamamoto method for indoxyl generation, several indoles were probed to evaluate the relative ratio of indirubin to indigo. Broadly, electron deficient indoles led to some indirubin formation with significant amounts of indigo in each instance. In contrast, more electron rich systems produced indigo as the sole product. Experimental results supported the hypothesis that indirubin formation proceeded through a condensation pathway between the keto and enol tautomers of indoxyl, which contrasts with the free radical pathway likely needed to generate indigo. To support further understanding for this reaction, a detailed evaluation was undertaken for the molybdenum-catalysed generation of indoxyl.

## Results

### Temperature effects

**Scheme 2:**
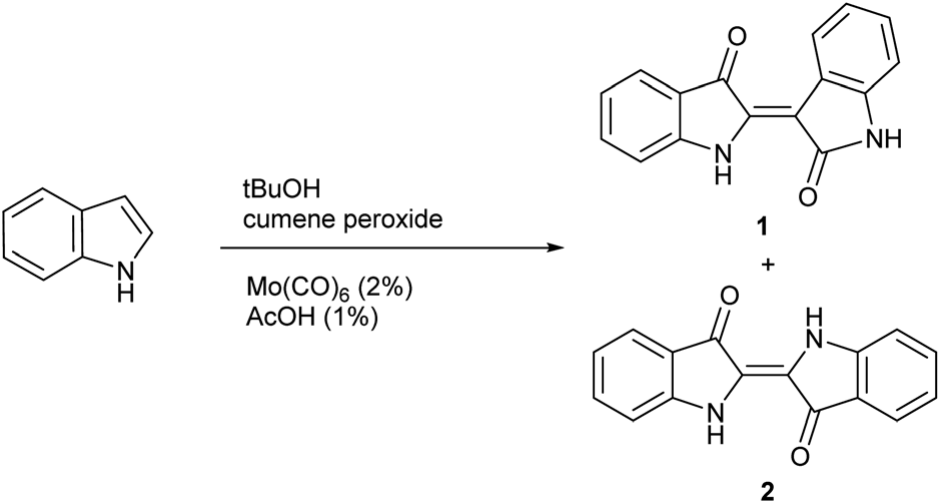
General procedure for the synthesis of 1 and 2.

While general experimental conditions for indoxyl generation were evaluated in significant detail for the Yamamoto method, the role of temperature was not extensively explored. With divergent mechanisms likely present, it was considered that temperature could be a key variable to tune the reaction towards either indigo or indirubin products and ultimately lead to the development of a new synthetic method. To evaluate this possibility, indole was subjected to previously determined reaction conditions while varying temperature as shown in Scheme 2 to form 1 and 2. The results from this study are shown in Table 1 at 86 °C (reflux), 60 °C, 40 °C and ambient temperature. For each reaction, the ratio of indirubin-to-indigo is indicated as determined by ^1^H NMR as well a percent recovery, which constitutes the combined yield of both products, which have the same molecular weight. Recovered starting material was not isolated for each trial as it was removed in the work-up procedure. Although indole only led to 2 at 86 °C, the amount of 1 can be increased significantly at lower temperatures with a 3 : 2 ratio of indirubin-to-indigo (60% indirubin) formed at ambient temperature.

**Table 1:** Condensation of 1 with 2 at variable temperatures

Temp. (°C)	Time (hours)	Indirubin : indigo	Recovery^a^ (%)
86	24	0 : 1	79
60	48	2 : 5	72
40	120	1 : 1	72
23	672	3 : 2	41

a Recovery is defined as the combined yields of 1 and 2.

This temperature effect shows promise to tune selectivity from indigo to indirubin for the homodimerization of indoles under indoxyl-generating conditions, giving promise that a general procedure for the synthesis of indirubins from indigo can be developed.

### Substituent effects

To further probe this observation, an array of indoles was evaluated with various substituent types and positions. Utilizing Scheme 2 to ensure consistency in the comparison of results, each substrate was subjected to three temperatures for a set period based upon the general reactivity of indole observed in the previous experiment. The room-temperature reactions were omitted as they were not practical due to the time needed for execution.

#### Assay of electron deficient systems

Table 2 summarizes the series of reactions performed on electron deficient indoles relative to the parent system with substituents located at the 5, 6 and 7 positions. From this series, some general trends have emerged. Product ratios of indirubin to indigo improves with decreasing temperature with nearly exclusive indirubin formation in most instances when the group is located at the 5 or 7 position. One notable example is 5,5′-dinitroindirubin (5), which only left a trace of indigo at 40 °C. The results for the 6-position are not as promising, but indirubin is still formed as the major product. An exception to this was observed for 6,6′-indirubin dicarboxylic acid (13), which produced a small amount of indirubin that was intractable from the parent indigo. In contrast, no product was seen for the reaction of 6-azaindole, even when performed at elevated temperatures (120 °C), which is likely due to an unfavorable interaction with the molybdenum catalyst.

**Table 2:** Ratio of indirubin to indigo for electron deficient indoles at variable temperatures. Reactions were run at 5 mmol scale with ratios determined by ^1^H NMR integration. Recovery is defined as the additive yield of indirubin and indigo unless otherwise noted

Indole	Indirubin	Indigo	86 °C for 24 hours		60 °C for 48 hours		40 °C for 120 hours	
			Indirubin : indigo	Recovery (%)	Indirubin : indigo	Recovery (%)	Indirubin : indigo	Recovery (%)
H	1	2	0 : 1	79	2 : 5	72	1 : 1	72
5-NO_2_^a^	5	6	8 : 3	66	>20 : 1	68	>20 : 1	22
5-CO_2_H	7	8	1 : 2	59	1 : 3	67	2 : 1	55
5-CN	9	10	2 : 3	62	2 : 1	52	7 : 2	42
6-NO_2_	11	12	2 : 9	67	2 : 1	71	3 : 1	39
6-CO_2_H	13	14	0 : 1	89	0 : 1	98	1 : 10	86
6-Aza	—	—	0 : 0	NR	0 : 0	NR	0 : 0	NR
7-CHO	15	16	b	65	>20 : 1	33	1 : 0	4
7-Aza	17	—	1 : 0	78	1 : 0	46	1 : 0	19

a 5,5′-Dinitroindirubin was characterized through formation of its corresponding 3′-oxime (35).

b The reaction mixture was complex, containing 15 as the major product, 16 as a minor product, and numerous unidentified products.

In comparison to other published methods, significant functional group tolerance is demonstrated as the reaction produces indirubin in the presence of numerous compounds that are typically sensitive to competing methodologies. While not the best yielding, 5,5′-indirubin dicarboxylic acid (7) forms as the major product. Moreover, this product was isolated by chromatography with a mixture of ethyl acetate and methanol spiked with a few drops of trifluoroacetic acid. In contrast, 7,7-diformylindirubin (5) was the only product at 40 °C and was nearly pure when run at 60 °C, constituting the first example of a formyl-substituted indirubin. There were other instances where indirubin formation was confirmed, but due to insolubility of the corresponding indigo in DMSO and other solvents, these data were not included in this assay.‡‡Dimethyl indirubin 5,5′-dicarboxylate, dimethyl indirubin 5,5′-dicarboxylate and 6,6′-dicyanoindirubin.

#### Assay of electron rich systems

A series of electron rich indoles were subjected to the same reaction conditions, noting that in the initial publication, only indigo was observed for the two examples run at 86 °C, 2 and 5,5′-dimethoxy indigo (20). Table 3 summarizes the results of these systems compared to the parent system. In many cases, significant quantities of indirubin were produced, but not with the same selectivity observed for the electron deficient systems. Nonetheless, indirubin constituted over 50% of product for all successful reactions of this type at 40 °C. Direct comparison of substitution site effects shows a slight preference for the 6 position over the 5 and 7 positions, counter to the selectivity seen for electron poor systems.

**Table 3:** Ratio of indirubin to indigo for electron rich indoles at variable temperatures. Reactions were run at 5 mmol scale with ratios determined by ^1^H NMR integration. Recovery is defined as the additive yield of indirubin and indigo unless otherwise noted

Indole	Indirubin	Indigo	86 °C for 24 hours		60 °C for 48 hours		40 °C for 120 hours	
			Indirubin : indigo	Recovery (%)	Indirubin : indigo	Recovery (%)	Indirubin : indigo	Recovery (%)
H	1	2	0 : 1	79	2 : 5	72	1 : 1	72
1-Me	—	—	0 : 0	NA	0 : 0	NA	0 : 0	NA
5-Me	18	19	0 : 1	73	2 : 3	73	8 : 5	42
5-OMe	—	20	0 : 1	65	0 : 1	46	0 : 1	53
5-OH	—	—	NR	NA	NR	NA	NR	NA
6-Me	21	22	1 : 2	76	3 : 2	49	4 : 1	30
6-OMe	—	23	0 : 1	28	0:0^a^	NA	0 : 0^a^	NA
7-Me	24	25	1 : 5^b^	77	2 : 1	69	3 : 1	46

a A complex array of products was observed leading to >20 unique methoxy peaks in the ^1^H NMR.

b An approximate 1 : 1 ratio of 24: 7,7′-dimethylisoindigo was also present during only this higher temperature run.

From this group, some limits were also observed. Namely, there were a series of unreactive indoles. For the reactions ran with 5-hydroxy indole, starting material was recovered, likely due to interactions with the molybdenum catalyst as was observed for 6-azaindole. In contrast, no products or starting materials were obtained for the reaction with 1-methyl indole, a surprising result which may suggest that after oxidation, decomposition occurred. Similarly, 6-methoxy indole, which produced the corresponding 6,6′-dimethylindigo (23) exclusively at 86 °C, gave a complex and inseparable mixture of many products, including the indigo as elucidated from the NMR for reactions performed at lower temperatures. Curiously, 5-methoxy indole yielded only 20, even at 40 °C. This reaction was repeated at room temperature and produced a small amount of indirubin (1 : 12 ratio) as observed by ^1^H NMR.

#### Assay of halogeno systems

Halogen-substituted indirubins that have been prepared and studied extensively by others as precursors are more readily available for the traditional Baeyer approach. Table 4 summarizes the results from this work from this assay with promising results. Notably, halogen-substituted indoles produced indirubin with near exclusivity in most cases, with the worst results seen for 6-bromoindole at a 14 : 1 ratio at 40 °C. The lone exception is for 4-chloroindole, which only produced indigo as expected due to steric constrains at the 4-position in proximity to the 3′ carbonyl.

**Table 4:** Ratio of indirubin to indigo for halogen substituted indoles at variable temperatures. Reactions were run at 5 mmol scale with ratios determined by ^1^H NMR integration. Recovery is defined as the additive yield of indirubin and indigo

Indole	Indirubin	Indigo	86 °C for 24 hours		60 °C for 48 hours		40 °C for 120 hours	
			Indirubin : indigo	Recovery (%)	Indirubin : indigo	Recovery (%)	Indirubin : indigo	Recovery (%)
H	1	2	0 : 1	79	2 : 5	72	1 : 1	72
4-Cl	—	26	0 : 1	58	0 : 1	64	0 : 1	50
5-Cl	27	28	4 : 1	84	10 : 1	88	>20 : 1	73
5-Br	29	30	20 : 1	66	>20 : 1	82	1 : 0	56
6-Cl	31	32	0 : 1	80	3 : 2	74	1 : 0	37
6-Br	33	34	2 : 1	82	10 : 1	73	14 : 1	70

## Discussion

The goal of this research was to utilize information from an earlier publication to test for a thermodynamic break between two competing pathways. The results strongly support that indigo synthesis is inhibited at lower temperatures, with the break point for this shift occurring somewhere between 40° and 60 °C in most cases. There is nuance in the effect of substituents for this reaction: electron deficient indoles work well, with a preference to effective, but show some preference to the 6 positon, whereas halegeno indoles work well at all positions. Even for the example where indirubin was not isolated, 6-methoxyindole, indigo formation was disrupted. This observation is consistent with a reaction (in its rate determining step) that occurs in a stepwise fashion, as opposed to a free-radical mechanism implicated for the formation of indigo.^17^

One competing hypothesis that was considered is that the reaction was dependent upon the rate of formation of indoxyl, a secondary result of lowering the temperature. A series of control reactions were performed for the parent system with variation of the concentration (more or less solvent) and catalyst load. The results were within parity with the original data from ^1^H NMR analysis (ranging from 33–50% indirubin), indicating that this is not the explanation for this result as the results skewed slightly in the other direction as the control reaction at 40 °C gave 50% indirubin.

Temperature-dependent interconversion between indirubin and indigo was tested as a competing mechanism by subjecting a pure sample of 1 to 1.1 equivalent of peroxide with 1% Mo(CO)_6_ in *t*-BuOH at 86 °C for 24 h. After workup, no conversion to 2 was observed by ^1^H NMR. Similarly, a pure sample of 2 ran at the lower temperature of 60 °C showed no interconversion after 24 h.

Deeper exploration of the mechanistic underpinnings of this system will require a different method of generation for indoxyl. Utilizing Yamamoto's method for indoxyl generation is convenient for making this an applicable synthesis, but it is not as conducive to a deeper evaluation as certain substrates are limited, likely through poisoning the catalyst. There is also a temperature limiting effect for indoxyl formation which is evident by trends in reduced yield and unreacted starting material. Since the temperature break for some systems may be lower, there is still opportunity to improve the ratio of indirubin production at the cost of synthetic simplicity if a new method amenable to lower temperatures is identified.

## Conclusions

Broadly, the results of this assay demonstrate that indirubin is synthesized selectively over indigo at reduced temperatures, in particular for electron deficient or halogen-substituted compounds. This method is broadly applicable as indoles are readily available as starting materials, both commercially and through synthetic means. Moreover, these reactions were performed at a robust 5 mmol scale, and have been successfully performed on a 100 mmol scale.^15^ Combined with the low cost nature of the other reagents such as the 80% cumyl peroxide, a side product of phenol production, this process has potential to be applied in a cost effective manner. It also is complimentary to the other contemporary homodimerization approach utilizing potassium borohydride. The assay above provides a template for the targeted synthesis of homodimeric indirubins with different substitution patterns, with optimal balance of indirubin production *vs.* yield occurring on average at 60 °C and application of this method should leverage these data to anticipate optimal reaction conditions for a given substrate.

Research is ongoing in exploring other methods for generating indoxyl at low temperatures to further probe the limits of this method and to expand mechanistic understanding using a less complex system. Indoxyl silyl enol ethers have been identified for this purpose. Moreover, the use of isatin as a trap to access heterodimeric systems, which was demonstrated in principle in an earlier publication and is currently under development, will further compliment this approach.

## Experimental

### General methods

All starting materials were commercially available and used without additional purification from TCI, Aldrich, Fisher Scientific, Ambeed and Alfa Aesar. NMR spectra were determined in d_6_-DMSO at 300 MHz (^1^H NMR) on a Bruker Avance™ spectrometer or 400 MHz (^1^H NMR) on a JEOL JNM-ECZ400S spectrometer. The chemical shifts are expressed in *δ* values relative to the appropriate solvent. For reaction mixtures, NMR integration was used to assess approximate relative values using high scan number to optimize accuracy. IR spectra were obtained on a PerkinElmer Spectrum RX I with an ATR (Ge crystal) from Pike Technologies. The UV/Vis spectra were obtained on a Cary 50 using DMSO as the solvent. HRMS data on new compounds was independently contracted through the University of Iowa High Resolution Mass Spectrometry facility. All student researchers received appropriate safety training though the college prior to conducting research.

### Synthetic details

#### General procedure for the synthesis of indirubin

An indole (5 mmol) was added to a round bottom flask along with Mo(CO)_6_ (0.1 mmol), acetic acid (0.1 mmol) and *t*-butanol (10.9 mL). After stirring, 80% cumene peroxide in cumene (11 mmol) was added and the flask was allowed to stir under one of the three conditions (a) 86 °C for 24 h, (b) 60 °C for 48 h or (c) 40 °C for 120 h. The flask was allowed to cool to room temperature and was filtered through a Buchner funnel, washing the solid precipitate with cold methanol until the filtrate consistency was clear. The solid was placed into a clean round bottomed flask, dried under vacuum, and analysed by ^1^H NMR in DMSO-d_6_. The recovery constitutes the amount of indirubin and indigo combined is indicated in Tables 1–3 above unless otherwise noted. Starting material was removed during the washing process with methanol. For each condition as defined above, data are reported in the following format; weight (g), amount (mol) recovery (%) and ratio (indirubin-to-indigo).

#### Indirubin (1) and indigo (2)

Were synthesized by the general procedure above starting with indole to produce (a) 0.517 g, 1.97 mmol, 79%, 0 : 1; (b) 0.473 g, 1.80 mmol, 72%, 2 : 5; (c) 0.470 g, 1.79 mmol, 72%, 1 : 1. Spectroscopy: ^1^H NMR of 1 was previously published^8^^1^H NMR (300 MHz, d_6_-DMSO) *δ* – 11.03 (1H, s); 10.91 (1H, s); 8.78 (1H, d, *J* = 7.5 Hz); 7.66 (1H, d, *J* = 7.2 Hz); 7.59 (1H, t, *J* = 7.5 Hz); 7.43 (1H, d, 8.1 Hz); 7.27 (1H, t, *J* = 7.4 Hz); 7.01–7.07 (2H, m); 6.91 (1H, d, *J* = 7.8 Hz). ^1^H NMR of 2 was consistent with widely available spectra.^18^^1^H NMR (300 MHz, d_6_-DMSO) *δ* – 10.51 (2H, s); 7.62 (2H, d, *J* = 7.5 Hz); 7.52 (2H, t, *J* = 7.2 Hz); 7.34 (2H, d, *J* = 8.1 Hz); 6.96 (2H, t, *J* = 8.4 Hz).

#### 5,5′-Dinitroindirubin (5) and 5,5′-dinitroindigo (6)

Were synthesized by the general procedure above starting with 5-nitroindole to produce (a) 0.581 g, 1.65 mmol, 66%, 8 : 3; (b) 0.601 g, 1.71 mmol, 68%, >20 : 1; (c) 0.198 g, 0.56 mmol, 22%, >20 : 1. Spectroscopy and spectrometry: compound 5 was not previously published. An analytical sample was prepared and gave burgundy crystals m.p. (>350 °C, no decomposition observed); UV/Vis (DMSO) *λ*_max_ 545 nm; ^1^H NMR (400 MHz, d_6_-DMSO) *δ* – 11.74 (1H, s); 11.64 (1H, s); 9.67 (1H, d, *J* = 2.4 Hz); 8.45 (1H, dd, *J* = 8.4 Hz and 2.4 Hz); 8.42 (1H, d, *J* = 2.4 Hz); 8.22 (1H, dd, 8.4 Hz and 2.4 Hz); 7.59 (1H, d, *J* = 8.8 Hz); 7.08 (1H, d, *J* = 8.8 Hz); HRMS ESI (−) C_16_H_7_O_6_N_4_ 351.0372, theoretical 351.0371. Compound 6 was previously published.^19^^1^H NMR (400 MHz, d_6_-DMSO) *δ* – 11.47 (2H, s); 8.53 (2H, d, *J* = 2.8 Hz); 7.85 (2H, dd, *J* = 8.8 Hz and 2.4 Hz); 7.52 (2H, d, *J* = 8.8 Hz).

#### 5,5′-Dinitroindirubin-3′-oxime (35)

Was synthesized to confirm the structure for 5 due to low solubility preventing the procurement of a ^13^C NMR. To a round bottomed flask, 0.3365 g (0.955 mmol) of 5 was added along with 0.500 g hydroxylamine hydrochloride, 0.750 g of sodium acetate trihydrate and 10 mL of DMSO. The reaction mixture was allowed to stir for 22 hours at 100 °C. After the reaction vessel cooled, 30 mL of water was added, and the slurry was allowed to stir for 10 minutes. The product was filtered on a Buchner funnel and rinsed sparingly with a 2 mL of methanol. The product was transferred to a round bottomed flask and dried under vacuum to yield 0.251 g (0.683 mmol, 72%) of 35. Spectroscopy and spectrometry: compound 35 was not previously published. An analytical sample was prepared and gave brick red crystals m.p. (>350 °C, slight darkening of the solid); UV/Vis (DMSO) *λ*_max_ 504 nm; IR ^1^H NMR (400 MHz, d_6_-DMSO) *δ* – 14.36 (1H, s); 12.17 (1H, s); 11.49 (1H, s); 9.44 (1H, s); 8.97 (1H, s); 8.33 (1H, d, *J* = 8.8 Hz); 8.10 (1H, d, *J* = 8.4 Hz); 7.63 (1H, d, 8.8 Hz); 7.04 (1H, d, *J* = 8.8 Hz); ^13^C NMR (100 MHz, d_6_-DMSO) *δ* – 171.2, 150.6, 150.0, 146.6, 144.9, 142.2, 128.9, 128.8, 123.8, 123.0, 119.0, 116.9, 112.9, 109.5, 109.4, 100.6. IR (cm^−1^) 2145, 2672, 1672, 1618, 1572, 1518, 1488, 1330, 1294. HRMS ESI (−) C_16_H_8_O_6_N_5_ 366.0477 theoretical = 366.0480.

#### Indirubin 5,5′-dicarboxylic acid (7) and indigo 5,5′-dicarboxylic acid (8)

Were synthesized by the general procedure above starting with indole 5-carboxylic acid to produce (a) 0.512 g, 1.46 mmol, 59%, 1 : 2; (b) 0.589 g, 1.68 mmol, 67%, 1 : 3; (c) 0.478 g, 1.36 mmol, 55%, 2 : 1. Spectroscopy: ^1^H NMR was previously published for 7 (ref. 19) and 8.^21^ An analytical sample of 7 was prepared by running flash chromatography on a portion using 4 : 1 EtOAc : MeOH spiked with TFA as the eluent. For 7^1^H NMR (400 MHz, d_6_-DMSO) *δ* – 11.91 (2H, br s); 11.36 (1H, s); 11.28 (1H, s); 9.42 (1H, s); 8.10 (2H, overlapping s) and (d); 7.87 (1H, d, *J* = 8.0 Hz); 7.48 (1H, d, *J* = 8.4 Hz); 6.96 (1H, d, *J* = 8.0 Hz). For 8^1^H NMR (300 MHz, d_6_-DMSO) *δ* – 12.82 (2H, s); 11.03 (2H, s); 8.13 (2H, d, *J* = 1.5 Hz); 8.10 (2H, dd, *J* = 8.4 Hz and 1.5 Hz); 7.40 (2H, d, *J* = 8.4 Hz).

#### Indirubin 5,5′-dicarbonitrile (9) and indigo 5,5′-dicarbonitrile (10)

Were synthesized by the general procedure above starting with 5-cyanoindole to produce (a) 0.483 g, 1.55 mmol, 62%, 2 : 3; (b) 0.407 g, 1.30 mmol, 52%, 2 : 1; (c) 0.326 g, 1.04 mmol, 42%, 7 : 2. Spectroscopy: ^1^H NMR was previously published for 9 and 10.^22^ For 9, ^1^H NMR (300 MHz, d_6_-DMSO) *δ* – 11.57 (1H, s); 11.48 (1H, s); 9.05 (1H, d, *J* = 1.7 Hz); 8.21 (1H, d, *J* = 0.9 Hz); 8.01 (1H, dd, *J* = 8.4 Hz and 1.8 Hz); 7.75 (1H, dd, *J* = 8.4 Hz and 1.8 Hz) 7.60 (1H, d, *J* = 8.4 Hz). 7.08 (1H, d, *J* = 8.1 Hz). For 10^1^H NMR (300 MHz, d_6_-DMSO) *δ* – 11.20 (2H, s); 8.14 (2H, d, *J* = 1.2 Hz); 7.91 (2H, dd, *J* = 8.4 Hz and 1.5 Hz); 7.48 (2H, d, *J* = 8.1 Hz).

#### 6,6′-Dinitroindirubin (11) and 6,6′-dinitroindigo (12)

Were synthesized by the general procedure above starting with 6-nitroindole to produce (a) 0.592 g, 1.68 mmol, 67%, 2 : 9; (b) 0.639 g, 1.81 mmol, 73%, 2 : 1; (c) 0.346 g, 0.98 mmol, 39%, 3 : 1. Spectroscopy: ^1^H NMR was previously published for 11 and 12.^22^^1^H NMR for 11, (300 MHz, d_6_-DMSO) *δ* – 11.75 (1H, s); 11.36 (1H, s); 8.89 (1H, d, *J* = 8.7 Hz); 8.30 (1H, d, *J* = 2.1 Hz); 7.98 (1H, dd, *J* = 8.7 Hz and 2.1 Hz); 7.92–7.87 (2H, m); 7.64 (1H, d, *J* = 2.1 Hz). For 12^1^H NMR (300 MHz, d_6_-DMSO) *δ* – 11.20 (2H, s); 8.18 (2H, d, *J* = 1.8 Hz); 7.91 (2H, d, *J* = 8.1 Hz); 7.75 (2H, dd, *J* = 8.1 Hz and 1.8 Hz).

#### Indirubin 6,6′-dicarboxylic acid (13) and indigo 6,6′-dicarboxylic acid (14)

Were synthesized by the general procedure above starting with indole 6-carboxylic acid to produce (a) 0.777 g, 2.22 mmol, 89%, 0 : 1; (b) 0.861 g, 2.46 mmol, 98%, 0 : 1; (c) 0.692 g, 1.97 mmol, 86%, 1 : 10. A trace of 13 was syntehsized and was intractable from the reaction mixture and was not isolated. Spectroscopy: compound 14 was previously published.^21^ For 14^1^H NMR (300 MHz, d_6_-DMSO) *δ* – 13.26 (br s, 2H); 10.85 (2H, s); 7.95 (2H, s); 7.73 (2H, d, *J* = 8.1 Hz); 7.51 (2H, d, *J* = 8.1 Hz).

#### Indirubin 7,7′-dicarbaldehyde (15) and indigo 7,7′-dicarbaldehyde (16)

Were synthesized by the general procedure above starting with indole 7-carbaldehyde to produce (a) 0.516 g, 1.62 mmol, 66%, 8 : 5; (b) 0.263 g, 0.82 mmol, 33%, 18 : 1; (c) 0.033 g, 0.10 mmol, 4%, 1 : 0. Spectroscopy and spectrometry: Compound 15 was not previously published. An analytical sample was prepared and gave burgundy crystals m.p. (>350 °C, no decomposition observed); UV/Vis (DMSO) *λ*_max_ 536 nm; ^1^H NMR (400 MHz, d_6_-DMSO) *δ* – 11.77 (1H, s); 11.58 (1H, s); 10.14 (1H, s); 10.08 (1H, s) 8.97 (1H, d, *J* = 7.6 Hz); 8.17 (1H, d, *J* = 7.6 Hz); 7.99 (1H, d, *J* = 7.6 Hz); 7.75 (1H, d, 7.6 Hz); 7.25 (1H, t, *J* = 7.6 Hz); 7.23 (1H, t, *J* = 7.6 Hz). ^13^C NMR (100 MHz, d_6_-DMSO) *δ* – 194.1, 191.2, 187.4, 172.3, 150.4, 142.4, 141.3, 139.4, 131.1, 130.7, 130.5, 123.1, 122.2, 122.1, 121.1, 120.4, 119.4, 106.6. IR (cm^−1^) 3336, 1676, 1601, 1471, 1439, 1393, 1169, 1013. HRMS ESI (−) C_18_H_9_N_2_O_4_ 317.0568 theoretical = 317.0568; Compound 16 was previously published^21^ and tentatively assigned.

#### 7,7′-Diazaindirubin (17)

Was synthesized by the general procedure above starting with 7-azaindole to produce (a) 0.515 g, 1.95 mmol, 79%, 1 : 0; (b) 0.301 g, 1.14 mmol, 46%, 1 : 0; (c) 0.126 g, 0.48 mmol, 19%, 1 : 0. Spectroscopy: ^1^H NMR of 17 was previously published^20^^1^H NMR (300 MHz, d_6_-DMSO) *δ* – 11.60 (1H, s); 10.76 (1H, s); 8.86 (1H, d, *J* = 7.5 Hz); 8.52 (1H, dd, *J* = 5.1 Hz and 1.5 Hz); 8.13–8.18 (2H, m); 7.16 (1H, dd, 7.5 Hz and 5.1 Hz); 7.11 (1H, dd, *J* = 7.2 Hz and 5.4 Hz).

#### 5,5′-Dimethylindirubin (18) and 5,5′-dimethylindigo (19)

Were synthesized by the general procedure above starting with 5-methylindole to produce (a) 0.533 g, 1.84 mmol, 73%, 0 : 1; (b) 0.533 g, 1.84 mmol, 73%, 2 : 3; (c) 0.304 g, 1.05 mmol, 42%, 8 : 5. Spectroscopy: ^1^H NMR was previously published for 18 (ref. 14) and 19.^23^ For 18, ^1^H NMR (300 MHz, d_6_-DMSO) *δ* – 10.92 (1H, s); 10.78 (1H, s); 8.62 (1H, s); 7.45 (1H, s); 7.41 (1H, dd, *J* = 7.8); 7.31 (1H, d, *J* = 8.1 Hz); 7.08 (1H, d, *J* = 8.1 Hz). 6.79 (1H, d, *J* = 7.8 Hz); 2.33 (3H, s); 2.31 (3H, s). For 19^1^H NMR (300 MHz, d_6_-DMSO) *δ* – 10.35 (2H, s); 7.41 (2H, s); 7.35 (2H, d, *J* = 8.4 Hz); 7.22 (2H, d, *J* = 8.1 Hz) 2.30 (6H, s).

#### 5,5′-Dimethoxyindigo (20)

Was synthesized by the general procedure above starting with 5-methoxyindole. To produce (a) 0.521 g, 1.62 mmol, 65%, 1 : 0; (b) 0.437 g, 1.36 mmol, 53%, 1 : 0; (c) 0.369 g, 1.14 mmol, 46%, 1 : 0. Spectroscopy: ^1^H NMR was published for 20,^15^ and was previously made on multiple occasions.^24^ For 20^1^H NMR (300 MHz, d_6_-DMSO) *δ* – 10.27 (2H, s); 7.28 (2H, d, *J* = 9.0 Hz); 7.16 (2H, dd, *J* = 9.0 Hz and 2.7 Hz); 7.09 (2H, d, *J* = 2.4 Hz); 3.78 (6H, s).

#### 6,6′-DimethylIndirubin (21) and 6,6′-dimethylindigo (22)

Were synthesized by the general procedure above starting with 6-methylindole to produce (a) 0.549 g, 1.89 mmol, 76%, 1 : 2; (b) 0.353 g, 1.22 mmol, 49%, 3 : 2; (c) 0.217 g, 0.75 mmol, 30%, 6 : 1. Spectroscopy: compounds 21 and 22 were previously published.^25^ For 21^1^H NMR (300 MHz, d_6_-DMSO) *δ* – 10.84 (2H, s); 8.66 (1H, d, *J* = 8.1 Hz); 7.54 (1H, d, *J* = 7.8 Hz); 7.22 (1H, s); 6.84 (2H, two unresolved doublets, *J* = 7.8 Hz); 6.73 (1H, s); 2.37 (3H, s); 2.33 (3H, s) For 22^1^H NMR (300 MHz, d_6_-DMSO) *δ* – 10.35 (2H, s); 7.50 (2H, d, *J* = 8.1 Hz); 7.13 (2H, s); 6.77 (2H, d, *J* = 7.8 Hz) 2.36 (6H, s).

#### 6,6′-Dimethoxyindigo (23)

Was synthesized by the general procedure above starting with 6-methoxyindole to produce (a) 0.230 g, 0.69 mmol, 28%, 0 : 1; (b) 0.367 g, complex mixture; (c) 0.017 g, complex mixture. Compound 23 was previously published.^24^ For 23^1^H NMR (300 MHz, d_6_-DMSO) *δ* – 10.22 (2H, s); 7.48 (2H, d, *J* = 8.8 Hz); 6.82 (2H, d, *J* = 2.4 Hz); 6.46 (2H, dd, *J* = 8.8 Hz and 2.4 Hz); 3.80 (6H, s).

#### 7,7′-DimethylIndirubin (24) and 7,7′-dimethylindigo (25)

Were synthesized by the general procedure above starting with 6-methylindole to produce (a) 0.561 g, 1.93 mmol, 77%, 1 : 5 (and 1 : 5 with isoindigo); (b) 0.501 g, 1.73 mmol, 69%, 2 : 1; (c) 0.334 g, 1.15 mmol, 46%, 3 : 1. Spectroscopy: compounds 24 and 25 were previously published.^25^ For 21^1^H NMR (400 MHz, d_6_-DMSO) *δ* – 11.02 (1H, s); 10.54 (1H, s); 8.55 (1H, d, *J* = 8.0 Hz); 7.51 (1H, d, *J* = 7.2 Hz); 7.44 (1H, d, *J* = 7.2 Hz); 7.07 (1H, d, *J* = 7.2 Hz) 6.96 (1H, t, *J* = 7.6 Hz). 6.94 (1H, d, *J* = 8.0 Hz); 2.30 (3H, s); 2.22 (3H, s). For 22^1^H NMR (400 MHz, d_6_-DMSO) *δ* – 9.59 (2H, s); 7.46 (2H, d, *J* = 8.0 Hz); 7.37 (2H, d, *J* = 8.4 Hz); 6.91 (2H, t, *J* = 7.6 Hz) 2.35 (6H, s). 7,7′-Dimethylisoindigo was produced as a side product to the reaction.^26^^1^H NMR (400 MHz, d_6_-DMSO) *δ* −10.82 (2H, s); 8.86 (2H, d, *J* = 8.0 Hz); 7.13 (2H, d, *J* = 7.6 Hz); 8.84 (2H, t, *J* = 8.0 Hz).

#### 4,4′-Dichloroindigo (26)

Was synthesized by the general procedure above starting with 4-chloroindole to produce (a) 0.482 g, 1.46 mmol, 58%, 0 : 1; (b) 0.530 g, 1.61 mmol, 64%, 0 : 1; (c) 0.412 g, 1.25 mmol, 50%, 0 : 1. Spectroscopy: ^1^H NMR was previously published for 26.^27^^1^H NMR (400 MHz, d_6_-DMSO) *δ* – 10.81 (2H, s); 7.44 (2H, t, *J* = 7.6 Hz); 7.24 (2H, d, *J* = 7.6 Hz); 6.90 (2H, d, *J* = 6.8 Hz).

#### 5,5′-DichloroIndirubin (27) and 5,5′-dichloroindigo (28)

Were synthesized by the general procedure above starting with 5-chloroindole to produce (a) 0.692 g, 2.10 mmol, 84%, 4 : 1; (b) 0.727 g, 2.20 mmol, 88%, 10 : 1; (c) 0.605 g, 1.83 mmol, 53%, >20 : 1. Spectroscopy: ^1^H NMR was previously published for 27 (ref. 14) and 28.^19^ For 27^1^H NMR (300 MHz, d_6_-DMSO) *δ* – 11.22 (1H, s); 11.05 (1H, s); 8.79 (1H, d, *J* = 1.8 Hz); 7.70 (1H, d, *J* = 1.8 Hz) 7.65 (1H, dd, *J* = 8.7 Hz and 2.1 Hz); 7.47 (1H, dd, *J* = 8.7 Hz and 2.1 Hz); 7.32 (1H, d, *J* = 8.4 Hz) 6.92 (1H, d, *J* = 8.4 Hz). For 28^1^H NMR (300 MHz, d_6_-DMSO) *δ* – 10.73 (2H, s); 7.67 (2H, d, *J* = 2.4 Hz); 7.57 (2H, dd, *J* = 8.4 Hz and 1.8 Hz); 7.18 (2H, d, *J* = 8.1 Hz).

#### 5,5′-DibromoIndirubin (29) and 5,5′-dibromoindigo (30)

Were synthesized by the general procedure above starting with 5-bromoindole to produce (a) 0.691 g, 1.65 mmol, 66%, 20 : 1; (b) 0.861 g, 2.05 mmol, 82%, >20 : 1; (c) 0.590 g, 1.41 mmol, 56%, >20 : 1. Spectroscopy: ^1^H NMR was previously published for 29 (ref. 14) and 30.^19^ For 29^1^H NMR (300 MHz, d_6_-DMSO) *δ* – 11.21 (1H, s); 11.05 (1H, s); 8.92 (1H, d, *J* = 1.8 Hz); 7.80 (1H, d, *J* = 1.5 Hz) 7.75 (1H, dd, *J* = 8.4 Hz and 2.1 Hz); 7.44 (1H, dd, *J* = 8.4 Hz and 1.8 Hz); 7.32 (1H, d, *J* = 8.1 Hz) 6.87 (1H, d, *J* = 8.1 Hz). For 30^1^H NMR (300 MHz, d_6_-DMSO) *δ* – 10.73 (2H, s); 7.76 (2H, s); 7.68 (2H, dd, *J* = 8.4 Hz and 1.8 Hz); 7.31 (2H, d, *J* = 8.4 Hz).

#### 6,6′-Dichloroindirubin (31) and 6,6′-dichloroindigo (32)

Were synthesized by the general procedure above starting with 6-chloroindole to produce (a) 0.659 g, 2.00 mmol, 80%, 0 : 1; (b) 0.613 g, 1.86 mmol, 74%, 3 : 2; (c) 0.282 g, 0.85 mmol, 37%, 1 : 0. Spectroscopy: ^1^H NMR was previously published for 31 and 32.^19^ For 31^1^H NMR (300 MHz, d_6_-DMSO) *δ* – 11.18 (1H, s); 11.10 (1H, s); 8.75 (1H, d, *J* = 8.7 Hz); 7.68 (1H, d, *J* = 8.1 Hz) 7.52 (1H, d, *J* = 1.5 Hz); 7.10 (1H, dd, *J* = 8.7 Hz and 1.8 Hz); 7.07 (1H, dd, *J* = 8.4 Hz and 1.8 Hz) 6.93 (1H, d, *J* = 2.1 Hz). For 32^1^H NMR (300 MHz, d_6_-DMSO) *δ* – 10.21 (2H, s); 7.48 (2H, d, *J* = 8.4 Hz); 6.81 (2H, d, *J* = 1.5 Hz); 6.76 (2H, dd, *J* = 8.1 Hz and 1.8 Hz).

#### 6,6′-DibromoIndirubin (33) and 6,6′-dibromoindigo (34)

Were synthesized by the general procedure above starting with 6-bromoindole to produce (a) 0.859 g, 2.05 mmol, 82%, 2 : 1; (b) 0.764 g, 1.82 mmol, 73%, 10 : 1; (c) 0.737 g, 1.76 mmol, 70%, 14 : 1. Spectroscopy: ^1^H NMR was previously published for 33 (ref. 14) and 34.^28^ For 33^1^H NMR (300 MHz, d_6_-DMSO) *δ* – 11.18 (1H, s); 11.08 (1H, s); 8.68 (1H, d, *J* = 8.4 Hz); 7.69 (1H, d, *J* = 1.8 Hz) 7.60 (1H, d, *J* = 8.4 Hz); 7.24 (1H, dd, *J* = 6.3 Hz and 2.1 Hz); 7.21 (1H, dd, *J* = 6.6 Hz and 1.8 Hz) 7.06 (1H, d, *J* = 1.8 Hz). For 34^1^H NMR (300 MHz, d_6_-DMSO) *δ* – 10.21 (2H, s); 7.57 (2H, d, *J* = 8.1 Hz); 7.00 (2H, d, *J* = 1.5 Hz); 6.85 (2H, dd, *J* = 8.1 Hz and 1.5 Hz).

## Author contributions

J. S.: conceptualization, data curation, formal analysis, funding acquisition, writing-original draft, project methodology, project supervision, visualization. K. K.; data curation, project supervision, investigation, supervision. K. W., M. V., J. C.; data curation, investigation, writing – review & editing. A. K., S. S., J. H.; data curation, investigation.

## Conflicts of interest

There are no conflicts to declare.

## Supplementary Material
